# The Osteopontin Level in Liver, Adipose Tissue and Serum Is Correlated with Fibrosis in Patients with Alcoholic Liver Disease

**DOI:** 10.1371/journal.pone.0035612

**Published:** 2012-04-18

**Authors:** Stéphanie Patouraux, Stéphanie Bonnafous, Cosmin S. Voican, Rodolphe Anty, Marie-Christine Saint-Paul, Maria-Alessandra Rosenthal-Allieri, Hélène Agostini, Micheline Njike, Nadége Barri-Ova, Sylvie Naveau, Yannick Le Marchand-Brustel, Pascal Veillon, Paul Calès, Gabriel Perlemuter, Albert Tran, Philippe Gual

**Affiliations:** 1 INSERM, U1065, Equipe 8 «Complications hépatiques de l'obésité», Nice, France; 2 Université de Nice-Sophia Antipolis, Faculté de Médecine, Nice, France; 3 Centre Hospitalier Universitaire de Nice, Hôpital de l'Archet, Département de Biologie, Nice, France; 4 Centre Hospitalier Universitaire de Nice, Hôpital de l'Archet, Département Digestif, Nice, France; 5 INSERM, U996, IPSIT, Clamart, France; 6 Univ. Paris-Sud, Faculté de Médecine Paris-Sud, Kremlin-Bicêtre, France; 7 AP-HP, Hôpital Antoine Béclère, Service d'hépato-gastroentérologie, Clamart, France; 8 Centre Hospitalier Universitaire de Nice, Hôpital de l'Archet, Département d'Immunologie, Nice, France; 9 Centre Hospitalier Universitaire d'Angers, Service d'Hépatologie, Angers, France; 10 Universitatea de Medicina si Farmacie Craiova, Craiova, Romania; Institute of Hepatology London, United Kingdom

## Abstract

**Background:**

Osteopontin (OPN) plays an important role in the progression of chronic liver diseases. We aimed to quantify the liver, adipose tissue and serum levels of OPN in heavy alcohol drinkers and to compare them with the histological severity of hepatic inflammation and fibrosis.

**Methodology/Principal Findings:**

OPN was evaluated in the serum of a retrospective and prospective group of 109 and 95 heavy alcohol drinkers, respectively, in the liver of 34 patients from the retrospective group, and in the liver and adipose tissue from an additional group of 38 heavy alcohol drinkers. Serum levels of OPN increased slightly with hepatic inflammation and progressively with the severity of hepatic fibrosis. Hepatic OPN expression correlated with hepatic inflammation, fibrosis, TGFβ expression, neutrophils accumulation and with the serum OPN level. Interestingly, adipose tissue OPN expression also correlated with hepatic fibrosis even after 7 days of alcohol abstinence. The elevated serum OPN level was an independent risk factor in estimating significant (F≥2) fibrosis in a model combining alkaline phosphatase, albumin, hemoglobin, OPN and FibroMeter® levels. OPN had an area under the receiving operator curve that estimated significant fibrosis of 0.89 and 0.88 in the retrospective and prospective groups, respectively. OPN, Hyaluronate (AUROC: 0.88), total Cytokeratin 18 (AUROC: 0.83) and FibroMeter® (AUROC: 0.90) estimated significance to the same extent in the retrospective group. Finally, the serum OPN levels also correlated with hepatic fibrosis and estimated significant (F≥2) fibrosis in 86 patients with chronic hepatitis C, which suggested that its elevated level could be a general response to chronic liver injury.

**Conclusion/Significance:**

OPN increased in the liver, adipose tissue and serum with liver fibrosis in alcoholic patients. Further, OPN is a new relevant biomarker for significant liver fibrosis. OPN could thus be an important actor in the pathogenesis of this chronic liver disease.

## Introduction

Excessive alcohol consumption is the third leading preventable cause of death in the United States [1]. Regular alcohol use can result in hepatic steatosis, which eventually progresses to steatohepatitis, fibrosis and cirrhosis. Up to 40% of patients with severe acute alcoholic hepatitis die within six months [1]. A better understanding of the molecular mechanisms leading to the progression of liver disease and the identification of new potential actors of the pathogenesis of alcoholic liver diseases (ALD) are thus important challenges. Modification of gut permeability, production of acetaldehyde, NADH and ROS, activation of Endoplasmic Reticulum stress, recruitment of immune cells into the liver and hepatocyte apoptosis may be key mechanisms of alcohol-induced liver injury [1], [2]. In line with the involvement of death of hepatocytes, we recently showed that serum markers of hepatocyte death and apoptosis are non invasive biomarkers of advanced fibrosis in patients with alcoholic liver disease [3]. Others factors involved in the pathogenesis of ALD can originate from extra-hepatic sites, particularly adipose tissue. The pioneers in this field have reported excess weight to be an independent risk factor for steatosis, acute alcoholic hepatitis (AAH) and cirrhosis in patients with ALD [4]; body mass index (BMI) is an independent risk factor for fibrosis in non-obese alcoholic patients [5] and high blood pressure, apolipoprotein A-1 concentration and BMI are predictive of steatosis [6]. More recently, Naveau *et al.* demonstrated that adipose tissue inflammation correlated with the severity of pathological features in the liver of alcoholic patients [7].

Recently, altered hepatic expression of osteopontin (OPN), a Th1 cytokine, chemokine and profibrogenic extracellular matrix protein, has been reported in an animal model of alcohol-induced liver injury [8], [9], [10] and in the liver of patients with alcoholic hepatitis and fibrosis [11], [12]. In female rats fed an ethanol containing Lieber-DeCarli liquid diet for 6 weeks and then challenged with LPS, the elevated circulating and hepatic levels of OPN play an important role in neutrophil accumulation and liver injury [8], [10], [13], [14]. OPN could also be involved in fibrogenesis. Indeed, OPN was upregulated in rodent liver fibrosis models [15], [16], [17] and in activated hepatic stellate cells [17], [18], [19]. OPN was required for myofibroblast differentiation [20] and upregulation of OPN expression in the liver of patients with ALD-cirrhosis was recently reported [12].

Similarly, OPN appears to play an important role in the occurrence of liver complications in a mouse model of non alcoholic fatty liver disease (NAFLD) [15], [21], [22], [23]. We also reported recently that the elevated expression of OPN was associated with adipose tissue macrophage accumulation and liver steatosis in humans [24] and evidence of OPN overexpression with progressive NAFLD has been confirmed recently [12].

Based on the evidence that OPN can be considered as a potential actor in alcohol-induced hepatic complications in rodents, we focused our study on serum, liver and adipose tissue OPN levels in patients with ALD and searched for a correlation with the disease severity. The evaluation of this marker in estimating significant fibrosis was also examined

## Materials and Methods

### Study population

**1) Alcoholic patients. Estimation group:** From October 1997 to June 1998, 109 consecutive heavy alcohol drinkers (82 males, 27 females, 47±1 years old) admitted to our Liver unit for detoxification and/or inpatient rehabilitation were entered into this study as previously described [3], [25], [26]. **Validation group:** 95 additional consecutive heavy alcohol drinkers (73 males, 22 females, 48±9 years old) were entered into this study from November 2006 to December 2009. All patients had consumed over 80 g ethanol per day for more than 5 years. All patients were negative for circulating hepatitis B surface antigen, hepatitis C virus, and human immunodeficiency virus. No patient had osteoarthritis or rheumatoid arthritis. Fasting blood samples were obtained and used to measure alanine amino transferase (ALT), aspartate aminotransferase (AST), gamma glutamyl transferase (γGT). A needle liver biopsy was performed in all patients by the transparietal approach. Biopsies were processed routinely and stained with hematoxylin-eosin-saffron and Sirius Red. The quality of biopsies was sufficient for interpretation [27]. The length of the liver biopsy was over 15 mm. Histopathological features were semi-quantitatively evaluated (**Table 1**): the grade of steatosis (0, <5%; 1, 5%–30%; 2, >30%–60%; 3, >60%) and stage of fibrosis (from 0, none, to 4, cirrhosis). The grading of hepatitic inflammation was done according to the Activity score of Orrego et al. [28]. Briefly, hepatic inflammation was determined by the presence (I1) or absence (I0) of hepatocellular necrosis and ballooning degeneration, alcoholic Mallory's hyaline bodies, associated with an inflammatory reaction. Sera and liver tissues were stored at −80°C until use. **Second gene group:** 38 consecutive heavy alcohol drinkers admitted to the Hepatogastroenterology Department of Antoine Béclère University Hospital, Clamart, France, due to alcoholism and ALD, were prospectively included into this study. All patients underwent ultrasound-guided liver biopsy at admission. One specimen of liver tissue was used for histological analysis and another was immediately frozen in liquid nitrogen and stored at −80°C for subsequent RNA extraction. Specimens were stained with hematoxylin-eosin-saffron, Masson's trichrome and picrosirius red. All biopsy samples were evaluated by a pathologist blind to the patient's clinical and biological data and to the duration of alcoholism and daily alcohol intake. Histopathological features were evaluated semi-quantitatively as described above (**Table 1**). Subcutaneous adipose tissue biopsies were performed at admission and after 7 days of abstinence from ethanol consumption. Adipose tissue samples were removed by needle aspiration as previously described [7]. **2) Patients with chronic viral hepatitis C.** 86 patients with chronic viral hepatitis C were included from June 1999 to May 2009. Anti-HCV antibodies and HCV RNA were detected in their serum. Selected patients had an available liver biopsy and some blood markers. Fasting blood samples were collected immediately before or no more than 12 months after liver biopsy. Exclusion criteria comprised additional causes of liver disease, particularly HBV and HIV infections, complicated cirrhosis, anti-fibrotic treatment in the previous 6 months, alcohol consumption of more than 30 g/day in the five years prior to inclusion. Liver biopsies were performed using Menghini's technique with a 1.4–1.6 mm diameter needle. Biopsy specimens were fixed in a formalin–alcohol–acetic solution and embedded in paraffin; 5-µm thick sections were then cut and stained with hematoxylin–eosin–saffron. Liver fibrosis was staged from F0 to F4 according to the Metavir staging system [29]. The diagnostic target, significant fibrosis, was defined as follows: F2+F3+F4. Readings were performed by independent, senior pathologists specialized in hepatology. These pathologists were blinded to blood tests. **3) Obese patients.** Obese patients were recruited through the Department of Digestive Surgery and Liver Transplantation, where they underwent bariatric surgery for morbid obesity (Nice and Paris hospitals). Bariatric surgery was indicated for these patients in accordance with the French Guidelines for surgery for obesity. Briefly, they had a BMI of 40 or 35 kg/m^2^ with at least one complication. Exclusion criteria were as follows: presence of a hepatitis B or hepatitis C virus infection, excessive alcohol consumption (>20 g/day), or another cause of chronic liver diseases (primary biliary cirrhosis, autoimmune hepatitis, Wilson disease, genetic hemochromatosis, or biliary disease). Fasting blood samples were obtained before surgery. Surgical liver biopsies were obtained at the time of bariatric surgery. Liver bridging fibrosis was assessed by sirius red staining and was classified into five stages as follows: absent, mild, significant (incomplete septa), advanced (with complete septa) and cirrhotic.

**Table 1:** Characteristics of the Estimation, Second Gene and Validation groups.

	Estimation group		Second gene group	Validation group
	Serum	Liver	Adipose tissue and liver	Serum
N (female/male)	109 (27/82)	34 (5/29)	38 (7/31)	95 (22/73)
Age (years)	47±1	47±1	47±1	49±1
Alcohol (g/day)	163±10	169±16	148±17	159±9
AST (IU/L)	95±10	98±13	152±31	98±8
ALT (IU/L)	70±15	61±9	98±15	76±7
AST/ALT	1.8±0.1	1.8±0.2	1.5±0.1	2±0.1
γGT (IU/L)	361±38	429±73	472±65	379±43
Fibrosis n(%)				
F0	16(15)	6(17.6)	9(23.7)	9(9.5)
F1	49(45)	14(41.2)	13(34.2)	38(40)
F3	15(14)	6(17.6)	7(18.4)	8(8.4)
F3	23(21)	3(8.8)	5(13.2)	39(41.1)
F4	6(6)	5(14.7)	4(10.5)	1(1.1)
Inflammation n(%)	26(24)	6(17.6)	12(31.6)	
Steatosis n(%)				
<5%	8(7)	1(2.9)	4(10.5)	
5–30%	62(57)	18(52.9)	10(26.3)	
>30–60%	27(25)	9(26.5)	12(31.6)	
>60%	12(11)	6(17.6)	12(31.6)	

Data are expressed as Means ± SEM or N (%). AST: aspartate amino-tranferase; ALT: alanine amino-transferase; γGT: Gamma Glutamyl Transpeptidase.

All subjects gave their informed written consent to participate in this research study according to French legislation regarding Ethics and Human Research (Huriet-Serusclat law, the “Comité Consultatif de Protection des Personnes dans la Recherche Biomédicale de Nice" (N°03.613)(DGS 2003/0395), the “Ethics committee of Bicêtre Hospital" and “Ethics committee of Centre Hospitalier Universitaire of Angers Hospital" (N°2010-01) approved this study).

### Circulating levels of OPN, Leptin and FibroMeter®

Circulating OPN levels were evaluated with an ELISA (R&D Systems, Lille, France), as described in the manufacturer's instructions. All samples were analyzed in duplicate, in random order and blinded to the clinical/pathological data. The CV (%) of intra-assay precision and inter assay precision ranged from 2.6 to 4 and 5.4 to 6.6, respectively. The minimum detectable dose of OPN ranged from 0.006 to 0.024 ng/mL. Serum was validated for use in this assay as indicated by the manufacturer. However, serum values are approximately 50% of the plasma values because of proteolytic cleavage by thrombin during the clotting process. Leptin was assessed in the serum by MILLIPLEX MAP Human Adipokine Panel A kit (HADK1-61K-A, Millipore) using a Luminex instrument (Luminex) according to manufacturer's recommendations. The FibroMeter® index of fibrosis was calculated using an algorithm including hyaluronate, the prothrombin index, α2-macroglobulin and platelets [30].

### Immunostaining

Liver biopsies were fixed in 4% buffered formol, paraffin embedded and sectioned at a thickness of 2 micron Slides were then dewaxed at pH = 6 using PT link (cycle :start at 65°C ,20′ at 95°C, back to 65°C), and washed for 5 minutes in Envision Flex Wash Buffer. Immunohistochemical staining was done using Autostainer Link, with anti-CD15 antibodies (347420 BD Biosciences) as the primary antibody, and diaminobenzidine (DAB) as activated chromogen. Nuclear counterstaining was done with Hematoxylin. CD15+ cells were counted in 2 or 3 portal spaces for each patient at 400× magnification. Counting was performed blindly by one observer.

### Real-time quantitative PCR analysis

Total RNA was extracted from human tissues using a RNeasy Mini Kit (Qiagen, Courtaboeuf, France). The samples were treated with Turbo DNA-free™ (Applied Biosystems, Courtaboeuf, France) or RNAse-free DNAse kit (Qiagen) following the manufacturer's protocols. The quality of the isolated RNA was determined using the Agilent 2100 Bioanalyser with RNA 6000 Nano Kit (Agilent Technologies, Massy, France). Total RNA (0.5 or 0.35 µg) was reverse-transcribed with the High Capacity cDNA Reverse Transcription Kit (Applied Biosystems, Courtaboeuf, France) or with the RT^2^ First Strand kit (SABiosciences) for the “estimation" and "second gene" groups, respectively. For the estimation group, real-time quantitative PCR was performed using the ABI PRISM 7500 Fast Real Time PCR System and FAM™ dyes (Applied Biosystems, Courtaboeuf, France) following the manufacturer's protocols in C3M Genomics facilities. The TaqMan® gene expression assays were purchased from Applied Biosystems: OPN, (Hs00167093_m1); TGFβ (Hs00171257_m1), RPLP0 (Hs99999902_m1) and 18S (Hs99999901_s1). For the second gene group, real-time quantitative PCR was performed using a custom-made PCR array (SABiosciences) in the LightCycler® 480 instrument (Roche) with the RT^2^ SYBR® Green qPCR Master Mix (SABiosciences) according to the manufacturer's instructions. Gene expression values were normalized to the value of the housekeeping gene *RPLP0* (Ribosomal Phosphoprotein Large P0) or *18S* and calculated based on the comparative cycle threshold Ct method (2^−ΔΔCt^) as previously described [24], [31].

### Statistical analysis

Statistical significance of the differential gene expression or circulating levels between the two study groups was determined using the non-parametric Mann-Whitney test with the ΔCt or amount for each group, respectively. The non-parametric Kruskal-Wallis test was used for the comparison of more than two study groups. *P*<0.05 was considered as significant. As a post-hoc correction for Kruskal-Wallis test, the Scheffe's test has been performed (data not shown). Correlations were analyzed using the Spearman's rank correlation test. Comparisons were done using the Chi^2^ test or Fischer's exact tests for nominal data and by two-sample *t* tests for continuous data. Multivariate analyses were performed using binary logistic regression with estimation of odds ratios (OR) and 95% confidence intervals (95%CI). Diagnostic performance was determined by constructing a “receiver-operating characteristic" (ROC) curve and calculating the area under the ROC (AUROC) curve for determination of patients with significant fibrosis (F≥2) for serum OPN. From these curves, the best cut-off values were established for OPN, which were the values that maximized the sum of the sensitivity and specificity to identify patient status. Statistical analyses were performed using NCSS 2007 software.

## Results

### Clinical Characteristics of Patients

Two cohorts of heavy alcohol drinkers were included in this study, which examines retrospective (estimation cohort) and prospective (validation cohort) data. In the estimation cohort (109 patients), 36% of the patients had moderate or severe steatosis (S≥2), 24% presented with hepatic inflammation, 14% with moderate fibrosis (F = 2) and 27% with advanced fibrosis (F≥3) (**Table 1**). The estimation (**Table 1**) and validation (95 patients) cohorts were similar in terms of age, gender, alcohol consumption, AST, AST/ALT, γGT and hepatic fibrosis. The serum OPN level was evaluated in all patients, while OPN gene expression was evaluated in the liver of 34 patients from the estimation group (**Table 1** **: Liver**) and in the liver and adipose tissue in 38 patients from the second group (**Table 1** **: Second gene group**).

### The serum and hepatic OPN level increased with liver fibrosis in patients with alcoholic liver diseases

**Figure 1:**
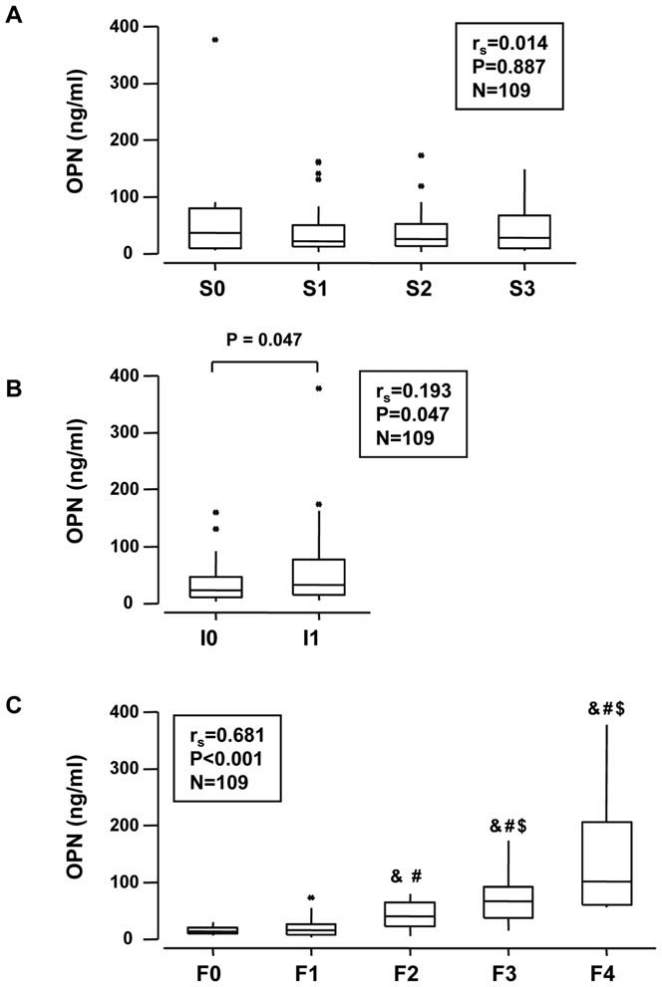
The serum OPN level correlated with fibrosis and steatohepatitis in 109 alcoholic patients (estimation cohort). The circulating levels of OPN were measured in the serum of 109 alcoholic patients (Table 1) and analyzed according to the grade of steatosis (S0, S1, S2, S3)(**A**), the presence of hepatic inflammation (I0, I1) (Hepatic inflammation was determined by the presence (I1) or absence (I0) of hepatocellular necrosis and ballooning degeneration, alcoholic Mallory's hyaline bodies, associated with an inflammatory reaction) (**B**) and the stage of fibrosis (F0, F1, F2, F3, F4)(**C**) (as described in Materials and Methods). Results were expressed as the median (25^th^, 75^th^ percentile). The Kruskal-Wallis test was used to compare the 4 groups S0, S1, S2 and S3, *P* = 0.900 and the 5 groups F0, F1, F2, F3 and F4, *P*<0.001. The Mann Whitney test compared A0 A1: *P* = 0.047. The Mann Whitney test compared the two groups (C): &, *P*<0.015, compared with F0; #, *P*<0.028, compared with F1; $, *P* = 0.007, compared with F2. Correlations between the serum OPN level with hepatic steatosis, hepatitis or fibrosis were analyzed using the Spearman's rank correlation test.

**Figure 2:**
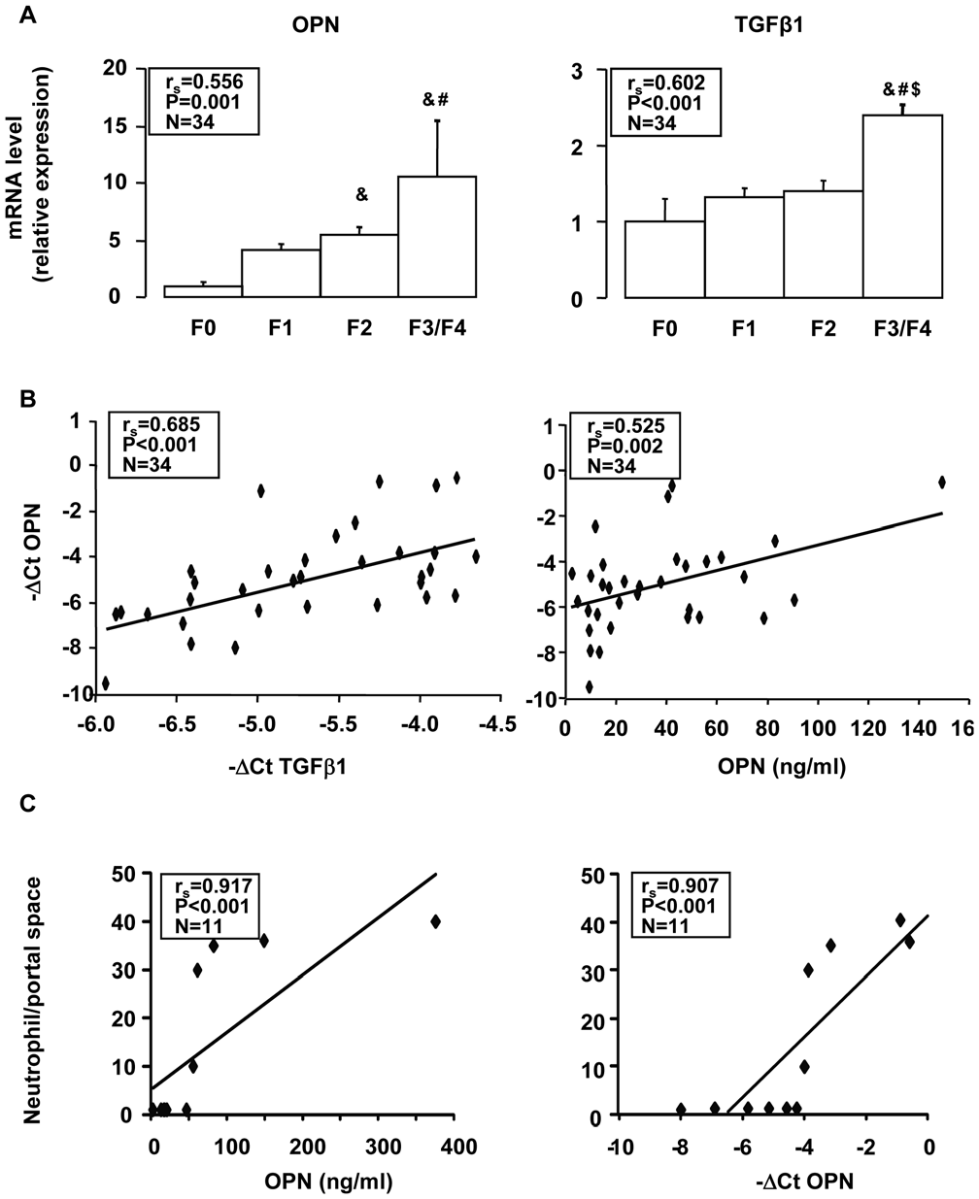
Hepatic OPN expression correlated with hepatic TGFβ expression, portal space neutrophils and the serum OPN level. **A**) Hepatic OPN and TGFβ1 expression levels were analyzed by real-time quantitative PCR in 34 alcoholic patients from the estimation group without (**F0**) (N = 6) or with mild (**F1**) (N = 14), moderate (**F2**) (N = 6) or advanced (**F3/4**) (N = 8) fibrosis. The mRNA levels were normalized to the mRNA levels of RPLP0. Results are expressed relative to the expression levels in F0 patients and expressed as means ± SEM. The Kruskal-Wallis test compared the 4 groups F0, F1, F2 and F3/F4: OPN, *P* = 0.037; TGFβ1, *P* = 0.002. Mann Whitney test compared the two groups: &, *P*≤0.011, compared with F0; #, *P*≤0.029, compared with F1; $, *P* = 0.008, compared with F2. **B**) Correlation between hepatic OPN gene expression (ΔCt) and either the hepatic TGFβ1 expression level (ΔCt) or the serum OPN level were analyzed using the Spearman's rank correlation test. **C**) Correlation between the number of portal space neutophils (Cf. Materials and methods) and serum OPN level or hepatic OPN gene expression (ΔCt) in 11 alcoholic patients (2F0/3F1/1F2/5F4) were analyzed using the Spearman's rank correlation test.

We first evaluated the serum OPN levels in the estimation cohort of 109 alcoholic patients. While hepatic steatosis had no effect on the serum OPN level (**Figure 1A**), the level was increased in patients with hepatic inflammation (I1) compared with patients without liver inflammation (I0) (**Figure 1B**) and was progressively increased in association with the stage of fibrosis (**Figure 1C**). The serum OPN concentrations of F0/F1 alcoholic patients (14 F0 and 51 F1) were comparable to those found in 16 lean subjects without diagnosed liver complications (BMI = 21±1 kg/m^2^) and in 14 morbidly obese patients (BMI = 43±1 kg/m^2^) with a minimal stage of fibrosis (4 F0 and 10 F1) and without hepatic steatosis, inflammation and ballooning (**Figure S1**). The serum OPN level further correlated strongly with fibrosis (r_s_ = 0.681, *P*<0.001) and, to a lesser extent with the hepatic inflammation (r_s_ = 0.193, *P* = 0.047). We then evaluated the hepatic expression level of OPN and TGFβ, a pro-fibrogenic factor [32], in 34 alcoholic patients from the estimation group (**Table 1**). Gene expression of OPN was increased in patients with moderate (F2) and advanced fibrosis (F3/F4) compared with patients with no fibrosis (F0) (**Figure 2A**). The liver OPN and TGFβ expression correlated with each other and with the grade of fibrosis (**Figures 2A and 2B**). The serum OPN level also correlated with its hepatic expression. Since OPN plays an important role in neutrophil accumulation and liver injury in the rat model of ASH [8], [10], [13], [14], we then evaluated OPN expression compared to the accumulation of neutrophil in the liver of patients with different grades of hepatic fibrosis (N = 11). The number of neutrophils in the portal space correlated with the circulating and hepatic OPN level (r_s_ = 0.917, *P*<0.001; r_s_ = 0.907, *P*<0.001; respectively) (**Figure 2C**). These results indicate that the circulating OPN level could reflect its hepatic expression and that there is a strong link between hepatic OPN expression and fibrogenesis.

### The adipose tissue OPN level increased with liver fibrosis in patients with alcoholic liver diseases

Since adipose tissue inflammation correlated with the severity of pathological features in the liver [7], we evaluated OPN expression in 38 ongoing alcoholic patients in both liver and adipose tissue biopsies (BMI = 24±4 kg/m^2^) (**Table 1** **: Second gene group**). In adipose tissue, the OPN level was already increased in patients with mild fibrosis (F1) compared with patients without liver fibrosis (3.10±0.51 fold increase, *P* = 0.0082) and correlated with hepatic fibrosis but not with liver steatosis and inflammation (**Table 2**). OPN level in adipose tissue did not correlate with BMI (r_s_ = 0.073, *P* = 0.689, N = 37) and the leptin level in adipose tissue (r_s_ = 0.138, *P* = 0.415, N = 37) or serum (r_s_ = 0.099, *P* = 0.577, N = 34). Expression in the liver correlated with fibrosis, as found in the estimation group (**Figure 1**), and with steatosis and inflammation (**Table 2**). Because recent alcohol intake could influence these results, the adipose tissue OPN expression was evaluated after 7 days of alcohol abstinence. Its expression still correlated with liver fibrosis (**Table 2**). Since OPN is mainly expressed by the inflammatory and immune cells in adipose tissue [22], [24], the adipose tissue of alcoholic patients could be inflamed at the early stage of liver fibrogenesis.

**Table 2:** Correlation between hepatic and adipose tissue OPN expression and hepatic steatosis, inflammation and fibrosis in alcoholic patients (the second gene group).

OPN expression in	Steatosis			Inflammation			Fibrosis		
	r_s_	P	N	r_s_	P	N	r_s_	P	N
Liver	0.432	0.007	38	0.712	<0.001	38	0.766	<0.001	38
Adipose tissue	0.077	0.652	37	0.117	0.492	37	0.361	0.028	37
Adipose tissue after 7 days of alcohol abstinence	0.063	0.722	34	0.170	0.337	34	0.342	0.048	34

Spearman's rank correlation test.

### The serum OPN level was an independent risk factor in estimating significant (F≥2) liver fibrosis in patients with alcoholic liver diseases

Since the circulating level of OPN was higher in patients with moderate fibrosis (F2) compared with patients with no or mild fibrosis (F0/F1) (**Figure 1C**), we investigated the pertinence of the serum OPN level for the estimation of significant fibrosis (F≥2). Using the univariate analysis (**Table 3**), patients with significant fibrosis (F≥2) were older and had a higher levels of OPN, γGT, alkaline phosphatase, bilirubin, hyaluronate and of the FibroMeter® index of fibrosis and lower levels of platelets, albumin, red blood cells, hemoglobin and of the prothrombin index compared to patients with mild fibrosis (F<2) (**Table 3**). In contrast, the gender, AST and ALT were not associated with F≥2. In a multivariate analysis including the OPN, alkaline phosphatase, albumin, hemoglobin and FibroMeter® level, OPN and FibroMeter® were the only independent variables when F≥2 was the judgment criterion (**Table 4**). Since FibroMeter® is a test that combines analysis for hyaluronate, the prothrombin index, α2-macroglobulin and platelets, we did not include these items in this analysis. The serum OPN level could thus be considered as an independent factor in estimating significant liver fibrosis in patients with alcoholic liver disease.

**Table 3:** Univariate analysis of the Estimation group according to the severity of the liver disease.

Data	F<2 (N = 65)	F≥2 (N = 44)	P
**Age (years)**	45.4±8.2	49.8±8.2	0.008279
**Gender (female/male)**	14/51	13/31	0,342053
**Platelets (10^9^/L)**	208.5±82.4	161.4±72.5	0.002715
**AST (IU/L)**	79.7±57.4	116.3±139.6	0.060470
**ALT (IU/L)**	62.0±45.1	82.1±240.2	0.512052
**γGT (IU/L)**	247.1±301.8	527.9±464.9	0.000221
**Bilirubin (µmol/L)**	9.8±5.97	56.2±94.6	0.000142
**Alkaline phosphatase (IU/L)**	85.2±37.7	142.7±68.2	<0.000001
**Red blood cells (10^12^/L)**	4.2±0.4	3.5±0.5	<0.000001
**Albumin (g/L)**	48.4±4.2	37.9±8.3	<0.000001
**Prothrombin index (%)**	98.4±3.9	75.9±22.4	<0.000001
**Hemoglobin (g/dL)**	13.8±1.6	11.9±1.7	<0.000001
**OPN (ng/mL)**	18.6±14.1	73.0±65.5	<0.000001
**Hyaluronate (µg/L)**	40.7±30.4	293.6±309.9	<0.000001
**FibroMeter**®	0.18±0.15	0.72±0.33	<0.000001

Patients were classified according to Fibrosis (F) <2 or ≥2.. Quantitative results are expressed as means ± standard deviations. AST: aspartate amino-tranferase; ALT: alanine amino-transferase; γGT: Gamma Glutamyl Transpeptidase; OPN: Osteopontin.

**Table 4:** Multivariate analysis of the Estimation group for the assessment of significant hepatic fibrosis.

Data	F≥2 (N = 44) versus F<2 (N = 65)		
	P	OR	95% CI
**Alkaline phosphatase**	0.925	0.999	0.9842–1.0143
**Albumin**	0.684	0.962	0.8018–1.1559
**Hemoglobin**	0.269	0.778	0.4987–1.2143
**OPN**	0.033	1.044	1.0033–1.0865
**FibroMeter**®	0.001	59.405	4.7129–748.7968

Patients were classified according to Fibrosis (F)<2 or ≥2. Multivariate analysis was realized using logistic regression.

### The serum OPN levels estimated significant fibrosis in alcoholic patients

**Figure 3:**
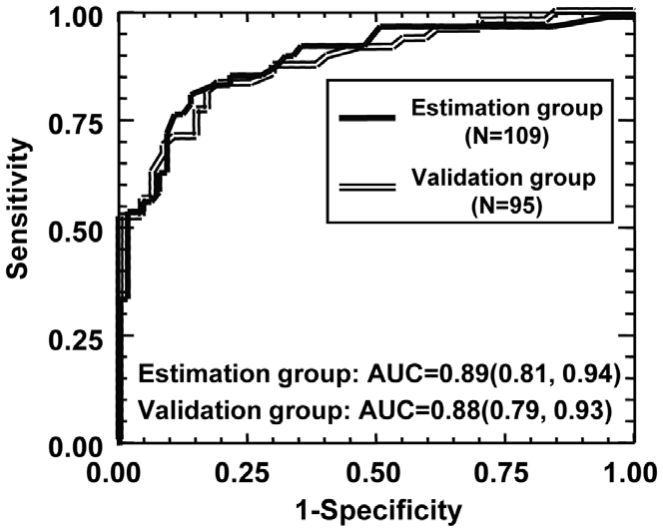
Levels of serum OPN for diagnosis of significant fibrosis (F≥2) in cohorts of alcoholic patients. The area under the ROC curves are shown for the performance of serum OPN levels in evaluating significant fibrosis (F≥2) in estimation (109 patients) and validation (95 patients) cohorts.

As shown in **Figure 3**, the area under the ROC curve in the estimation and validation cohorts for discrimination between F<2 and significant (F≥2) fibrosis with the serum OPN level was 0.89 (95% CI: 0.81–0.94) and 0.88 (95% CI: 0.79–0.93), respectively. Thus, the retrospective (estimation cohort) and prospective (validation cohort) studies led to the same result. Several cut-off values were calculated for the serum OPN level for the prediction of F≥2 in the estimation group (**Table S1**). For example, the cut-off point at 29 ng/mL estimated F≥2 with a sensitivity of 84%, a specificity of 81%, a Positive and Negative Predictive Value of 75.5% and 88.3%, respectively. To validate the performance of OPN in estimating significant fibrosis, we compared the OPN to the FibroMeter® level, a specific blood test using four biomarkers to predict fibrosis in ALD [30], and hyaluronate, serum cell death (Cytokeratin 18 total) and apoptotic markers (caspases generated cytokeratin 18 fragment), single biomarkers reflecting increased fibrogenesis [3], [33]. The area under the ROC curve in the estimation cohort for discriminating F<2 from significant (F≥2) fibrosis with the FibroMeter®, hyaluronate, cell death marker and apoptotic marker was 0.90 (95% CI: 0.82–0.95), 0.88 (95% CI: 0.79–0.94), 0.83 (95% CI: 0.73–0.89) and 0.74 (95% CI: 0.63–0.82), respectively. While OPN was more efficient than the apoptotic marker in estimating significant fibrosis (OPN AUROC versus serum apoptotic marker AUROC: *P* = 0.0011), OPN, FibroMeter®, hyaluronate and cell death marker levels estimated significant fibrosis with the same accuracy (OPN AUROC versus FibroMeter® AUROC: *P* = 0.752; versus hyaluronate AUROC: *P* = 0.791, versus cell death marker AUROC: *P* = 0.100) indicating that OPN should be an important actor or manifestation of liver fibrosis induced by chronic alcohol consumption.

### The serum OPN levels correlated with hepatic fibrosis and estimated significant (F≥2) fibrosis in patients with chronic viral hepatitis C

**Figure 4:**
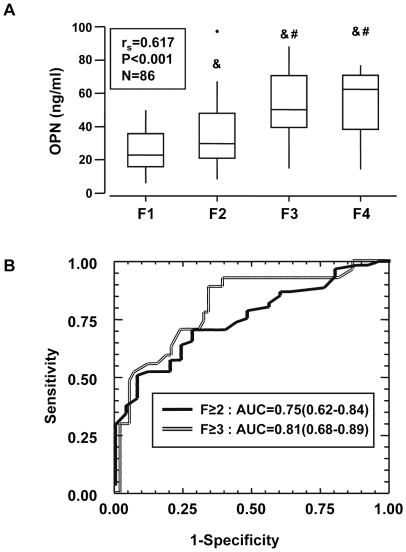
Levels of serum OPN in patients with chronic viral hepatitis C. (**A**) The circulating levels of OPN were measured in the serum of 86 patients with chronic hepatitis C (25 F1, 34 F2, 19 F3, 8 F4) and analyzed according to the stage of fibrosis. Results were expressed as the median (25^th^, 75^th^ percentile). The Kruskal-Wallis test was used to compare the 4 groups F1, F2, F3 and F4: *P*<0.001. The Mann-Whitney test compared the two groups: &, *P*≤0.048, compared with F1; #, *P*≤0.026, compared with F2. Correlation between the serum OPN level with hepatic fibrosis was analyzed using the Spearman's rank correlation test. (**B**) The areas under the ROC curves are shown for the performance of serum OPN levels in estimating significant fibrosis (F≥2) or advanced fibrosis (F≥3) in this cohort.

To explore whether the previous findings were specific to ALD, we evaluated the serum OPN levels in 86 patients with chronic viral hepatitis C including 25 patients with mild fibrosis (F1), 34 patients with moderate fibrosis (F2), 19 patients with severe fibrosis (F3) and 8 patients with cirrhosis (F4). The OPN level was increased in patients with moderate, severe fibrosis or cirrhosis compared with patients with mild fibrosis (F1) (**Figure 4A**). The serum OPN level further correlated strongly with fibrosis (r_s_ = 0.617, *P*<0.001) (**Figure 4A**) and, to a lower extent with hepatic inflammation (r_s_ = 0.360, *P* = 0.047). However, the serum OPN level in patients with chronic viral hepatitis C and significant fibrosis (F≥2) was lower than in alcoholic patients with significant fibrosis (F≥2) (HCV group: OPN median = 43.2 ng/mL, N = 61; Alcoholic group (estimation cohort): OPN median = 59.5 ng/mL, N = 44; Mann-Whitney test: *P* = 0.005). As shown in **Figure 4B**, the area under the ROC curve in patients with chronic viral hepatitis C to estimate significant (F≥2) and advanced (F≥3) fibrosis with the serum OPN level were 0.75 (95% CI: 0.62–0.84) and 0.81 (95% CI: 0.68–0.89), respectively. This indicated that circulating OPN could be a conserved response to chronic liver injury induced, *per se*, by alcohol and HCV.

## Discussion

We report here for the first time that the serum OPN level correlated with hepatic inflammation and fibrosis in heavy alcohol drinkers. The hepatic OPN expression level strongly correlated with hepatic neutrophils accumulation, the pro-fibrogenic factor TGFβ and hepatic fibrosis. Expression of OPN in adipose tissue correlated with hepatic fibrosis reinforcing the concept that adipose tissue was also inflamed in ALD. The serum level of OPN was an accurate independent factor in estimating significant liver fibrosis in both the estimation and validation cohort.

OPN expression detected in adipose tissue and in the liver of alcoholic patients was enhanced by the presence of hepatic fibrosis. The relative contribution of these two tissues in circulating OPN is difficult to estimate. While hepatic OPN expression correlated with the serum OPN level in our patients, further studies will be required to determine the relative role of these two tissues in modulating circulating OPN concentrations. Although the cellular origin of the increased hepatic OPN expression has not been precisely determined, increased OPN gene expression has been reported in macrophages, Kupffer cells, stellate cells, biliary epithelial cells and in inflammatory cells of the necrotic areas in rodent liver fibrosis models [15], [16], [18]. The observation that hepatic inflammation was associated with elevated serum OPN levels is in favor of a role for OPN in liver inflammation. Recent studies have highlighted the role of OPN in inflammatory liver diseases such as alcoholic and non alcoholic liver diseases and T cell-mediated hepatitis [8], [10], [13], [15], [23], [34], [35], [36], [37]. In rodent models of alcoholic liver diseases, neutrophils accumulation in the liver was mediated by OPN [8], [10], [13]. In agreement with these reports, we showed in a small number of patients that hepatic and systemic OPN expression correlated with neutophil infiltration in the portal space. OPN also facilitated the infiltration and accumulation of macrophages at sites of injury during the initial steps after carbon tetrachloride intoxication [16]. The recruitment and activation of the inflammatory and immune cells by OPN could enhance hepatic inflammation, which in turn may activate hepatic stellate cells and fibrogenesis. In agreement with this mechanism, the invalidation of the OPN gene decreased hepatic inflammation and consequently fibrosis in mice fed a choline-methione-deficient diet [12], [15]. Furthermore, it has been suggested that OPN could have fibrogenic properties. OPN expression was increased in activated hepatic stellate cells [17], [18], [19] and was required for myofibroblast differentiation [20]. OPN was regulated by the hedgehog pathway and directly promoted pro-fibrogenic responses [12]. The upregulation of OPN has been reported in the liver of patients with ALD-cirrhosis but also with NASH-cirrhosis, primary biliary cirrhosis, autoimmune hepatitis and primary sclerosing cholangitis, which suggested that OPN induction is a conserved response to chronic liver injury [12]. Excess weight was as an independent risk factor for fibrosis and cirrhosis in patients with ALD [4], [5]. More recently, Naveau *et al.* demonstrated that adipose tissue inflammation correlated with the severity of pathological features in the liver [7]. The increase in the production of OPN in adipose tissue could result from the activation of adipose tissue macrophages by LPS and cytokines such as TNFα and IL6. The latter were strongly increased in serum and adipose tissue of heavy alcohol drinkers [1], [7]. OPN plays an important role in the infiltration and accumulation of macrophages in the early stages of obesity. Indeed, we recently reported that elevated OPN expression in adipose tissue paralleled with adipose tissue macrophage infiltration and both phenomena were reversed after weight loss in obese patients [24]. While further investigation is necessary to determine the molecular mechanism of adipose tissue inflammation, adipose tissue OPN expression could lead to enrichment of adipose tissue in macrophages and to increased adipose tissue inflammation. The OPN level in adipose tissue could be related more to inflammation than adipose tissue gain. Indeed, OPN expression in adipose tissue did not correlate with BMI, leptin expression and leptinemia.

We have shown for the first time that the serum OPN level was an independent factor that estimates significant fibrosis in two independent cohorts of patients with ALD. A larger group of alcoholic patients with all the data allowing to repeat the univariate and multiple regression modeling will be done in the future. Since circulating OPN could increase in obese individuals and that obesity is also associated with a higher propensity to fibrosis and progression of chronic liver diseases, we analyzed 60 alcoholic patients with available BMI to determine an independent association between OPN and fibrosis. In these patients (39% with a significant fibrosis (F≥2)), serum OPN correlated with hepatic fibrosis (r_s_ = 0.497, *P*<0.001, N = 60) but not with BMI (r_s_ = 0.128, *P* = 0.328, N = 60). Using the univariate analysis, OPN but not BMI was associated with F≥2 (**Table S2**). In a multivariate analysis including the OPN, γGT and BMI, OPN was the only independent variable when F≥2 was the judgment criterion (**Table S3**). The serum OPN level could thus be considered as an independent factor estimating significant liver fibrosis. However, additional validations on a large number of alcoholic patients, controls subjects and morbidly obese group included patients with the full range of liver fibrosis with available BMI will be required. Liver biopsy remains the gold standard for assessment of liver fibrosis. However, problems with liver biopsy include sampling error and inter-observer variability. Fibroscan and the currently available blood-algorithm tests (Fibrotest®, FibroMeter®) or direct biomarker (Hyaluronate) can differentiate between mild and advanced disease [2], [33], [38], [39]. In the case of borderline results two or more methods can be combined. However, identification of novel markers is needed to improve blood-algorithm tests leading to quantification of fibrosis and to monitor the dynamic nature of fibrosis.

Interestingly, the OPN and FibroMeter® levels were the only independent variables when the F≥2 was the judgment criterion in a multivariate analysis including OPN, alkaline phosphatase, albumin, hemoglobin and FibroMeter® levels. Furthermore, OPN, FibroMeter® and hyaluronate estimated significant fibrosis with the same accuracy. OPN was a good marker in both retrospective (AUROC = 0.89 (0.81, 0.94)) and prospective (AUROC = 0.88 (0.79, 0.93)) studies. Moreover, the serum OPN levels accurately estimated advanced fibrosis (F≥3: AUROC = 0.91 (0.83, 0.95)) and cirrhosis (F = 4: AUROC = 0.91 (0.80, 0.96)) in alcoholic patients (from the estimation group).

Circulating levels of OPN were also modified in patients with liver complications related to the hepatitis C and B viruses. An elevated circulating OPN level was an excellent predictor of cirrhosis in patients with a hepatitis B infection [40]. The circulating levels of OPN were also modified in patients with liver complications induced by a hepatitis C infection. Indeed, we report here that the serum OPN in patients with chronic viral hepatitis C correlates with hepatic fibrosis, as previously reported for plasma of hepatitis C virus infected subjects [41]. OPN enhanced tumor development and metastases, since OPN was highly expressed in hepatocellular carcinoma (HCC) and correlated with worse prognosis [42]. However, the role of OPN in these diseases is still poorly understood. It was recently proposed that OPN could be a potential target for the control of HCC [43], [44].

In summary, circulating levels of OPN estimate, with good accuracy, significant fibrosis in heavy alcohol drinkers. Hepatic OPN also correlated with serum OPN and with hepatic inflammation, neutrophils infiltration, fibrosis and TGFβ expression. Infiltration of liver by parenchymal neutrophils is a prominent feature of Alcoholic Hepatitis [45]. Up-regulation of OPN in liver could contribute to this infiltration and the severity of Alcoholic Hepatitis. Multiple factors stimulate parenchymal and nonparenchymal cells to produce this chemokine including LPS, IL6 and TNFα. Adipose tissue of alcoholic patients was further inflamed, as evaluated by OPN expression. OPN adipose tissue level also correlated with hepatic fibrosis. Since TNFα and IL6 were strongly increased in adipose tissue and serum of heavy alcohol drinkers [1], [7], cytokines secreted by adipose tissue could enhance the inflammation and up-regulation of OPN in liver. The recruitment and activation of the inflammatory and immune cells by OPN could also enhance hepatic inflammation, which in turn may activate hepatic stellate cells and fibrogenesis. Furthermore, it has been suggested that OPN could have fibrogenic properties [12], [15], [16], [17], [18], [19], [20]. Studies focusing on the role of OPN in liver and adipose tissue function will be appropriate approaches to acquire more insight into the pathogenesis of human ALD.

## Supporting Information

Figure S1 **The serum OPN level in lean, obese and alcoholic patients with minimal stage (F0–F1) of fibrosis.** The circulating levels of OPN were measured in the serum of 16 lean subjects (**Lean**) without diagnosed liver complications (BMI = 21±1 kg/m^2^); of 14 morbidly obese patients (**Obese**) (BMI = 43±1 kg/m^2^) with a minimal stage of fibrosis (4 F0 and 10 F1) and without hepatic steatosis, inflammation and ballooning; and of 61 alcoholic patients (**Alcoholic**) with a minimal stage of fibrosis (14 F0 and 51 F1). Results were expressed as the median (25^th^, 75^th^ percentile). The Kruskal-Wallis test was used to compare the 3 groups *P* = 0.55.
(TIF)

Table S1 **Osteopontin levels forsignificant fibrosis (F≥2) assessment in the Estimation group.**
(DOCX)

Table S2 **Univariate analysis of 60 patients from Validation group according to the severity of liver disease.**
(DOCX)

Table S3 **Multivariate analysis in 60 patients from Validation group for the estimation of significant hepatic fibrosis.**
(DOCX)
