# Supplementary figures and images for: The Osteopontin Level in Liver, Adipose Tissue and Serum Is Correlated with Fibrosis in Patients with Alcoholic Liver Disease
Source: PLoS One. 2012 Apr 18;7(4):e35612. doi: 10.1371/journal.pone.0035612 (PMC3329460; doi:10.1371/journal.pone.0035612)

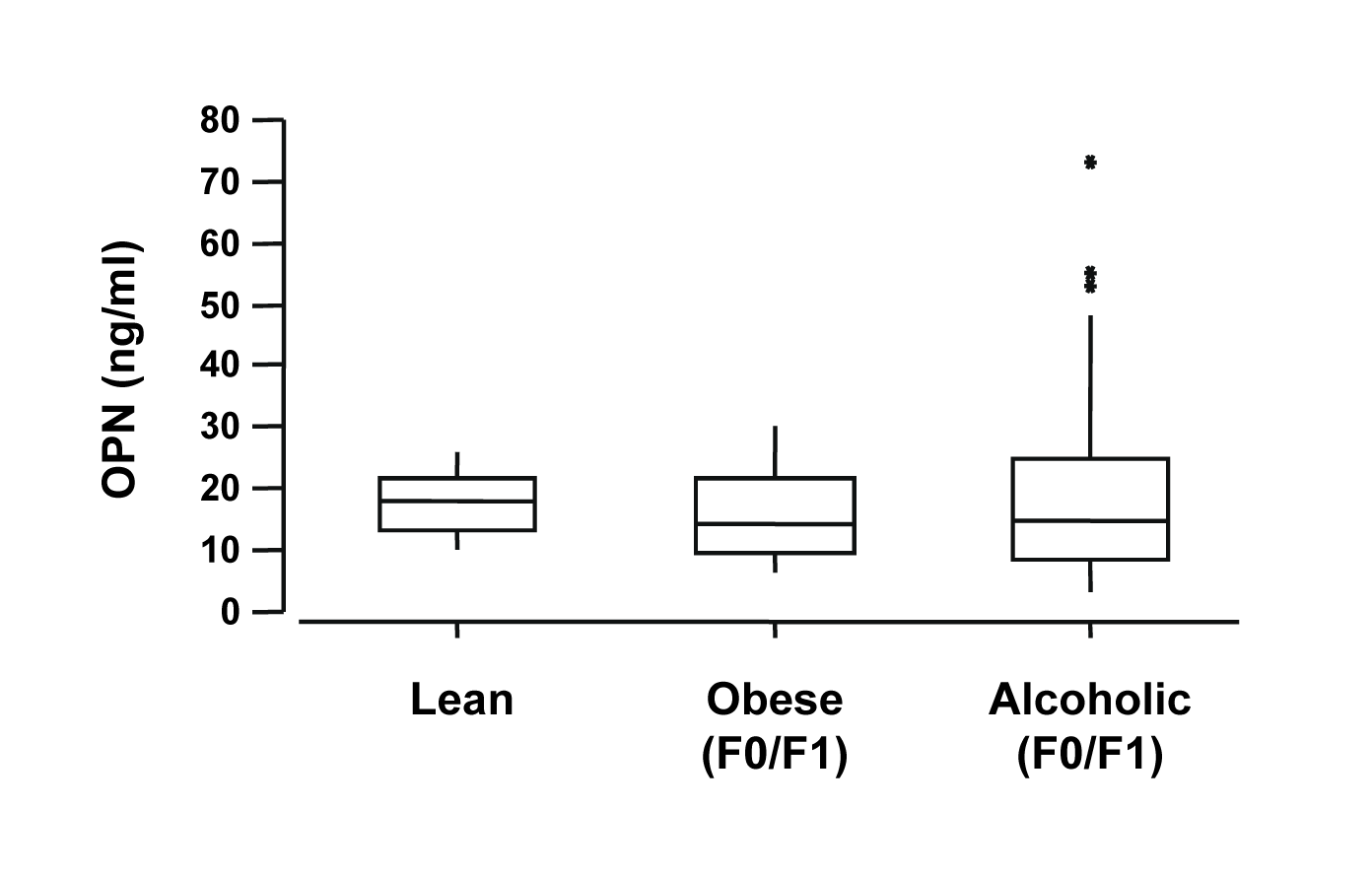

Supplement: Figure S1 — The serum OPN level in lean, obese and alcoholic patients with minimal stage (F0–F1) of fibrosis. The circulating levels of OPN were measured in the serum of 16 lean subjects (Lean) without diagnosed liver complications (BMI = 21±1 kg/m2); of 14 morbidly obese patients (Obese) (BMI = 43±1 kg/m2) with a minimal stage of fibrosis (4 F0 and 10 F1) and without hepatic steatosis, inflammation and ballooning; and of 61 alcoholic patients (Alcoholic) with a minimal stage of fibrosis (14 F0 and 51 F1). Results were expressed as the median (25th, 75th percentile). The Kruskal-Wallis test was used to compare the 3 groups P = 0.55. (TIF) [file pone.0035612.s001.tif]
