# Supplementary material for: The Osteopontin Level in Liver, Adipose Tissue and Serum Is Correlated with Fibrosis in Patients with Alcoholic Liver Disease
Source: PLoS One. 2012 Apr 18;7(4):e35612. doi: 10.1371/journal.pone.0035612 (PMC3329460; doi:10.1371/journal.pone.0035612)
Supplement: Table S1 — Osteopontin levels forsignificant fibrosis (F≥2) assessment in the Estimation group. (DOCX) [file pone.0035612.s002.docx]

| **Table S1. Osteopontin levels for significant fibrosis (F≥2) assessment in the Estimation group.** | | | | | | | |  |  |  |  | |  | |  | |  | |  | |  | |  | |  | |  | |  | |  | |  | |  |  |  |
| --- | --- | --- | --- | --- | --- | --- | --- | --- | --- | --- | --- | --- | --- | --- | --- | --- | --- | --- | --- | --- | --- | --- | --- | --- | --- | --- | --- | --- | --- | --- | --- | --- | --- | --- | --- | --- | --- |
|  |  |  |  |  | |  |  | | |  | |  | |  | |  | |  | |  | |  | |  | |  | |  | |  | |  | |  |  |  |  |
| **Cut-off Value** | **Sensitivity** | **Specificity** | **Likelihood**  **Ratio** | **PPV**  **(Prev. 0.4)** | | **NPV**  **(Prev. 0.4)** |  | | |  | |  | |  | |  | |  | |  | |  | |  | |  | |  | |  | |  | |  |  |  |  |
| **OPN (ng/mL)** |  |  |  |  | |  |  | | |  | |  | |  | |  | |  | |  | |  | |  | |  | |  | |  | |  | |  |  |  |  |
| **15** | **0.977** | **0.492** | **1.925** | **0.566** | | **0.970** |  | | |  | |  | |  | |  | |  | |  | |  | |  | |  | |  | |  | |  | |  |  |  |  |
| **20** | **0.932** | **0.646** | **2.633** | **0.641** | | **0.933** |  | | |  | |  | |  | |  | |  | |  | |  | |  | |  | |  | |  | |  | | | | | |
| **29** | **0.841** | **0.815** | **4.555** | **0.755** | | **0.883** |  | | |  | |  | |  | |  | |  | |  | |  | |  | |  | |  | |  | |  | | | | | |
| **36** | **0.773** | **0.892** | **7.175** | **0.829** | | **0.853** |  | | |  | |  | |  | |  | |  | |  | |  | |  | |  | |  | |  | |  | | | | | |
|  |  |  |  |  | |  |  | | |  | |  | |  | |  | |  | |  | |  | |  | |  | |  | |  | |  | | | | | |
| PPV: Positive Predictive value, NPV: Negative Predictive value; Prev: Prevalence | | | | |  |  |  |  |  |  |  |  |  |  |  |  |  |  |  |  |  |  |  |  |  |  |  |  |  |  |  |  |  |  |  |  |  |
|  |  |  |  |  | |  |  | | |  | |  | |  | |  | |  | |  | |  | |  | |  | |  | |  | |  | | | | | |
|  |  |  |  |  | |  |  | | |  | |  | |  | |  | |  | |  | |  | |  | |  | |  | |  | |  | | | | | |
|  |  |  |  |  | |  |  | | |  | |  | |  | |  | |  | |  | |  | |  | |  | |  | |  | |  | | | | | |
|  |  |  |  |  | |  |  | | |  | |  | |  | |  | |  | |  | |  | |  | |  | |  | |  | |  | | | | | |
