# Supplementary material for: The Osteopontin Level in Liver, Adipose Tissue and Serum Is Correlated with Fibrosis in Patients with Alcoholic Liver Disease
Source: PLoS One. 2012 Apr 18;7(4):e35612. doi: 10.1371/journal.pone.0035612 (PMC3329460; doi:10.1371/journal.pone.0035612)
Supplement: Table S2 — Univariate analysis of 60 patients from Validation group according to the severity of liver disease. (DOCX) [file pone.0035612.s003.docx]

| **Table S2: Univariate analysis of 60 patients from Validation group according to the severity of liver disease.** | | | |
| --- | --- | --- | --- |
|  |  |  |  |
| **Data** | **F<2 (N=43)** | **F≥2 (N=17)** | **P** |
| **Age (years)** | 44.2 ± 7.8 | 49.3 ± 9.1 | 0.034043 |
| **Gender (female/male)** | 9/34 | 2/15 | 0.408354 |
| **Platelets (10^9^/L)** | 216.0 ± 66.5 | 295.6 ± 396.7 | 0.208681 |
| **AST (IU/L)** | 86.6 ± 76.4 | 122.5 ± 83.0 | 0.116856 |
| **ALT (IU/L)** | 95.2 ± 86.6 | 87.1 ± 58.7 | 0.724900 |
| **γGT (IU/L)** | 358.9 ± 336.0 | 706.0 ± 606.4 | 0.006378 |
| **Bilirubin (μmol/L)** | 9.5 ± 5.9 | 17.7 ± 15.1 | 0.003239 |
| **OPN (ng/mL)** | 35.6 ± 14.6 | 76.8 ± 54.1 | 0.000021 |
| **BMI (kg/m^2^)** | 24.4 ± 4.5 | 25.4 ± 5.2 | 0.450423 |
| Patients were classified according to Fibrosis (F) <2 or ≥2. Quantitative results are expressed as means ± standard deviations. AST: aspartate amino-tranferase; ALT: alanine amino-transferase; γGT: Gamma Glutamyl Transpeptidase; OPN: Osteopontin. | | | |
|  |  |  |  |
|  |  |  |  |
|  |  |  |  |
