# Supplementary material for: The Osteopontin Level in Liver, Adipose Tissue and Serum Is Correlated with Fibrosis in Patients with Alcoholic Liver Disease
Source: PLoS One. 2012 Apr 18;7(4):e35612. doi: 10.1371/journal.pone.0035612 (PMC3329460; doi:10.1371/journal.pone.0035612)
Supplement: Table S3 — Multivariate analysis in 60 patients from Validation group for the estimation of significant hepatic fibrosis. (DOCX) [file pone.0035612.s004.docx]

| **Table S3: Multivariate analysis in 60 patients from Validation group for the estimation of significant hepatic fibrosis.** | | | |
| --- | --- | --- | --- |
|  |  |  |  |
|  |  |  |  |
| **Data** | **F ≥2 (N=17) versus F <2 (N=43)** | | |
|  | **P** | **OR** | **95% CI** |
| **γGT** | 0.05717 | 1.00148 | 0.99995-1.0030 |
| **BMI** | 0.17226 | 1,10141 | 0.95877-1.26527 |
| **OPN** | 0.00609 | 1.04987 | 1.01399-1.08702 |
| Patients were classified according to Fibrosis (F) <2 or ≥2. Multivariate analysis was realized using logistic regression. | | | |
|  |  |  |  |
